# Fear of Missing Out Predicts Distraction by Social Reward Signals Displayed on a Smartphone in Difficult Driving Situations

**DOI:** 10.3389/fpsyg.2021.688157

**Published:** 2021-07-16

**Authors:** Jérémy Matias, Jean-Charles Quinton, Michèle Colomb, Alice Normand, Marie Izaute, Laetitia Silvert

**Affiliations:** ^1^Université Clermont Auvergne, CNRS, LAPSCO, Clermont-Ferrand, France; ^2^Université Grenoble Alpes, CNRS, Grenoble INP, LJK, Grenoble, France; ^3^CEREMA, Equipe Recherche STI, Agence de Clermont-Ferrand, Clermont-Ferrand, France

**Keywords:** distraction, Fear of Missing Out, smartphone, social reward, driving, fog

## Abstract

Smartphones are particularly likely to elicit driver distraction with obvious negative repercussions on road safety. Recent selective attention models lead to expect that smartphones might be very effective in capturing attention due to their social reward history. Hence, individual differences in terms of Fear of Missing Out (FoMO) – i.e., of the apprehension of missing out on socially rewarding experiences – should play an important role in driver distraction. This factor has already been associated with self-reported estimations of greater attention paid to smartphones while driving, but the potential link between FoMO and smartphone-induced distraction has never been tested empirically. Therefore, we conducted a preliminary study to investigate whether FoMO would modulate attentional capture by reward distractors displayed on a smartphone. First, participants performed a classical visual search task in which neutral stimuli (colored circles) were associated with high or low social reward outcomes. Then, they had to detect a pedestrian or a roe deer in driving scenes with various levels of fog density. The social reward stimuli were displayed as distractors on the screen of a smartphone embedded in the pictures. The results showed a significant three-way interaction between FoMO, social reward distraction, and task difficulty. More precisely, under attention-demanding conditions (i.e., high-fog density), individual FoMO scores predicted attentional capture by social reward distractors, with longer reaction times (RTs) for high rather than low social reward distractors. These results highlight the importance to consider reward history and FoMO when investigating smartphone-based distraction. Limitations are discussed, notably regarding our sample characteristics (i.e., mainly young females) that might hamper the generalization of our findings to the overall population. Future research directions are provided.

## Introduction

Drivers’ attentional failures are considered among the major contributing factors in road accidents (Klauer et al., 2006; Dingus et al., 2016). According to the taxonomy proposed by Regan et al. (2011), distraction occurs when the driver’s attention is focused on driving-irrelevant information, resulting in insufficient attention toward driving-related stimuli that are critical for safety. Besides, driver distraction might be caused by external information like billboards (e.g., Chattington et al., 2009; Bendak and Al-Saleh, 2010; Dukic et al., 2013; Belyusar et al., 2016; Gitelman et al., 2019) but also by in-vehicle devices (e.g., Strayer, 2015; Arexis et al., 2017; Noy et al., 2018). In the latter case, smartphones are considered as a game changing technology regarding driver distraction, due to their particularly strong prevalence in our societies, and daily activities (Srivastava, 2005; Strayer, 2015; Ward et al., 2017; Pew Research Center, 2018; CREDOC, 2019).

### State of the Art

Interacting with a smartphone^1^ (e.g., reading or writing a message) can quite obviously lead to visual/manual distraction that impairs drivers’ performance (Caird et al., 2008, 2014; Dumitru et al., 2018; Ortiz et al., 2018). But smartphones might also produce cognitive distraction with comparable effects on drivers’ performance and road safety (Strayer, 2015) although the driver’s eyes are on the roadway and his/her hands are on the steering wheel (Strayer et al., 2003; Strayer and Drews, 2007). Indeed, in laboratory settings, it has been demonstrated that receiving a notification on a smartphone significantly disrupted performance on attention-demanding tasks, even when the participants did not directly interact with their device (Stothart et al., 2015). Above the temporary attentional capture produced by the notification, the authors argued that it might place a new prospective memory demand (i.e., waiting to respond, willingness to do so promptly) that could give rise to task-irrelevant thoughts (see also Waddell and Wiener, 2014). In the same vein, because smartphones allow us to interact with social media and “infotainment” systems, others authors argued that their *mere presence* might serve as a constant reminder of the broader social network that is potentially available (Thornton et al., 2014). In their study, the mere presence of participants’ own phones on their desktops negatively affected performance on attentional tasks, even though no incoming call nor message reception occurred during data collection. Accordingly, such a constant reminder of social network availability might also trigger mind wandering (Thornton et al., 2014; Stothart et al., 2015). Conjointly, other authors claimed that smartphones could produce cognitive distraction by draining attentional resources out of the task for the purpose of attentional control (Ward et al., 2017). Cognitive resources would indeed be recruited to inhibit both attentional capture and the (resulting) distracting thoughts generated by one’s smartphone. Therefore, such resources would no longer be available for attentional tasks, leading to poorer performance (but see also Hartmann et al. (2020) for null effects of smartphone presence on short-term and prospective memory). In sum, beyond their potential for visual/manual distraction, smartphones may also produce cognitive distraction and drain attentional resources out of the on-going task. Therefore, understanding attentional capture by smartphones appears crucial to understand driver distraction.

Classically, selective attention models relied on the interplay between bottom-up (e.g., saliency) and top-down factors (e.g., task set) to predict attentional capture by irrelevant distractors (Corbetta and Shulman, 2002; Fecteau and Munoz, 2006; Theeuwes, 2010; Zelinsky and Bisley, 2015). However, a third factor named selection history has emerged in more recent models of selective attention (Awh et al., 2012; Belopolsky, 2015; Luck et al., 2021). Past episodes of attentional selection would endow some stimuli with a particular “value” (through explicit or implicit learning), leading to lingering biases that affect future selection episodes above and beyond bottom-up and top-down factors (Theeuwes, 2018). More particularly, one class of phenomena that belongs to selection history is related to *reward* history (Anderson, 2015; Failing and Theeuwes, 2018; Theeuwes, 2018). Indeed, many studies have shown that stimuli (previously) associated with reward outcomes could trigger attentional capture in spite of being neither salient nor relevant in the task at hand (e.g., Hickey et al., 2010; Anderson et al., 2011a, b, 2017; Bourgeois et al., 2015; Bucker et al., 2015; Le Pelley et al., 2015; Munneke et al., 2015; Pearson et al., 2015; Anderson, 2016; Failing and Theeuwes, 2017).

We contend that the influence of smartphones on attention falls into the scope of reward history, and more precisely, of *social* reward history. First, users actually perceive the high reward value of their smartphone (Wilmer and Chein, 2016; Johannes et al., 2019), with social aspects being of particular importance (Özcan and Koçak, 2003; Srivastava, 2005; Waddell and Wiener, 2014; Sherman et al., 2016; Johannes et al., 2019). Moreover, studies have shown that smartphone use generates positive reinforcement learning (Turel and Serenko, 2012; Chen et al., 2019) and relies on neural regions involved in reward processing, social cognition, and attention (Sherman et al., 2016). Additionally, a large body of work has shown how people are extremely attached to their smartphone, as revealed by symptoms of behavioral addiction to their device (e.g., Walsh et al., 2008; Cheever et al., 2014; Clayton et al., 2015). However, it is worth noting that individual differences (e.g., impulsivity, reward-seeking behavior) also play an important role in smartphones’ influence on behavior (Brown et al., 2021) as well as in reward-based distraction (see Bourgeois et al., 2016; Failing and Theeuwes, 2018). In this framework, Fear of Missing Out (FoMO) is an emerging factor that has been shown to be associated with problematic internet and smartphone use in several studies (e.g., Elhai et al., 2016; Dempsey et al., 2019; Rozgonjuk et al., 2020; Wolniewicz et al., 2020). FoMO is defined as the pervasive apprehension that others might be having rewarding experiences from which one is absent, and is characterized by the desire to stay continually connected with what others are doing (Przybylski et al., 2013). Individuals reporting high levels of FoMO are more likely to want to stay constantly connected with others and are, therefore, more likely to engage with social media and technology (Przybylski et al., 2013). Accordingly, they reckon they are more prone to react to push notifications whereas people with lower levels of FoMO would be more able to resist being distracted by such stimuli (Rozgonjuk et al., 2019). Additionally, some studies have revealed a positive correlation between FoMO and driver distraction (Przybylski et al., 2013; Brown et al., 2021), indicating that the greater the FoMO the more frequently participants declare paying attention to their smartphone and reading/sending messages while driving.

### Objective and Hypothesis

So far, studies that aimed at linking FoMO with distraction (e.g., Przybylski et al., 2013; Rozgonjuk et al., 2019; Al-Furaih and Al-Awidi, 2020; Brown et al., 2021) have relied exclusively on self-reported estimations of distraction. To our knowledge, no study has yet experimentally tested whether FoMO would predict the behavioral distraction elicited by a smartphone, notably in a driving context. Our main objective was therefore to put more directly to the test the influence of FoMO on smartphone-based distraction while driving. More precisely, our hypothesis was that FoMO would predict distraction by smartphones but only for information that have a high social reward value. Therefore, participants first performed a classic association phase (Anderson, 2016) in which the attentional selection of target neutral stimuli (i.e., colored circles with no particular selection history before the experiment) was paired with a high or low social reward (i.e., more or less positive social feedback manipulated through different ratios of neutral/smiling faces). Subsequently, they performed a visual search task on pictures of driving scenes containing a target (i.e., a child pedestrian or a roe deer) that had to be accurately and quickly identified. Simultaneously, stimuli previously associated with social reward could appear as distractors on a smartphone, irrelevantly displayed in one corner of the pictures, as if put on the car dashboard, next to the wheel (e.g., as when used as a GPS device). Previous studies have reported that cognitive distraction triggered by a smartphone mainly occurred during attentional-demanding tasks (Thornton et al., 2014; Stothart et al., 2015). Accordingly, considering attention as a limited resource process (Kahneman, 1973; Shiffrin and Schneider, 1977), performance deficits due to smartphone distraction might be minimal with simple tasks that can be done with little or no attentional resources. However, greater attentional demands placed on the primary task should increase the potential for performance deficits. Consequently, we manipulated the task difficulty by increasing the fog density on the driving scenes as contrast reduction (Liu et al., 2009), so that prolonged evidence accumulation from visual inputs to reduce decision-making uncertainty (Quétard et al., 2015; Quétard, 2018), would increase attentional demands.

Thereafter, we first describe the main methodological aspects of our study, including the material used (i.e., FoMO scale, pictures …) and the procedure followed by the volunteers. Note that the methodological details for the reward association phase can be found as Supplementary Material. Then, we provide the statistical analyses of the behavioral results for both the association phase and the driving visual search task. Finally, theoretical implications and limitations of the present preliminary study are discussed and future research directions are provided.

## Materials and Methods

### Participants

Twenty-nine college students (seven males; *M* = 20 years old, *SD* = 2, min = 17, and max = 26) from the Université Clermont Auvergne were recruited in exchange of course credits. Volunteer sampling among psychology students explains why the sex ratio in our sample was biased toward female participants. All participants were right-handed and all reported normal or corrected-to-normal visual acuity, as well as normal color perception. They were naive to the experiment purpose but, prior to their participation, they were told that a 10€ gift card could be earned according to their performance. The study was ethically approved by an institutional review board (IRB00011540-2018-12).

### Apparatus

The participants were tested individually in a quiet room with constant ambient illumination, in front of a 14-inch VGA monitor (1,024 × 1,280 resolution, 60 Hz) at a distance of approximately 50 cm. The presentation of the stimuli, timing operations and data collection were controlled by E-Prime 2.0 software (Psychology Software Tools, Pittsburgh, PA, United States).

### Materials and Procedure

After giving their informed consent, the participants first completed the FoMO scale and then performed the association phase, followed by the driving visual search task. They were not explicitly informed about the role of the association phase nor about its relation with the following driving visual search task. The two tasks were presented as independent of each other. The experiment took approximately 60 min.

#### Fear of Missing Out Scale

The volunteers completed the French translation (see Michot et al., 2016) of the 10-items FoMO scale (Przybylski et al., 2013; see Supplementary Figure 1). Possible responses to the affirmations (e.g., “I fear my friends have more rewarding experiences than me”) ranged from 1 (“Not at all true for me”) to 5 (“Perfectly true for me”). The scale produces an average score ranging from 1 to 5, with higher scores indicating higher levels of FoMO.

#### Social Reward Association Phase

**FIGURE 1:**
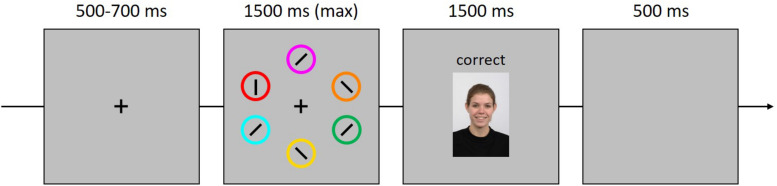
Example trial in the social reward association phase. Participants reported with a keypress the orientation of the bar within targets defined by their color (e.g., red in this case). Correct responses were followed by a feedback consisting of the presentation of a neutral or happy face (here, happy) from the Radboud Faces database (Langner et al., 2010). One target color was associated with a greater probability of a happy face vs. a neutral face (80–20%), while for the other target color this ratio was reversed.

The social reward association task was adapted from a classic visual search task (Anderson, 2016) in which the correct selection of different targets (i.e., circles of different colors) led to different social reward outcomes through the presentation of neutral or happy faces displayed in each trial with the performance feedback (see Figure 1). Because only minor changes were made to its original version (Anderson, 2016), the materials and procedure of the association phase are available as Supplementary Material.

#### Driving Visual Search Task

**FIGURE 2:**
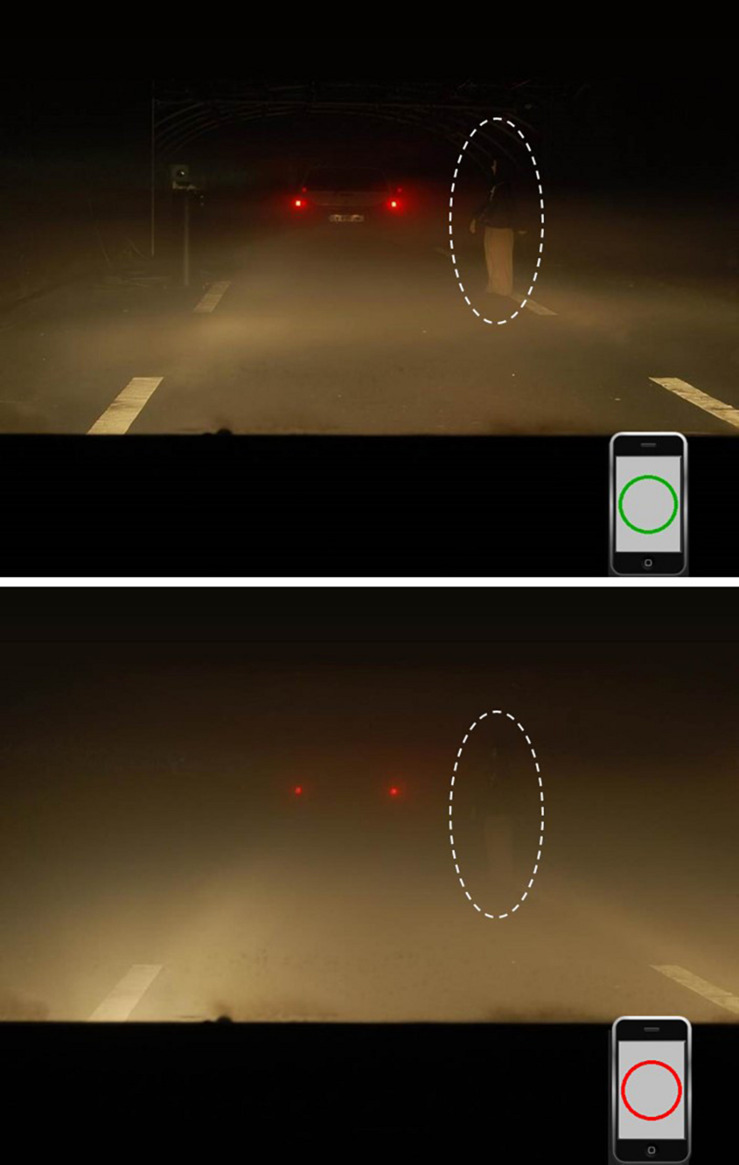
Examples of driving scene pictures used in the driving visual search task. Participants were required to identify as fast as possible the target (e.g., a child pedestrian, surrounded here by a dotted line for clarity, or a roe deer, not represented here) with pictures taken under low **(top)** or high fog density **(bottom)**. In 75% of the trials, an irrelevant low (e.g., green – **top**) or high (e.g., red – **bottom**) social reward distractor could appear on the screen of a smartphone embedded in each picture (the smartphone displayed an empty gray screen in the remaining trials - not represented here).

All stimuli were displayed on a black background. A central gray fixation cross (192, 192, 192) preceded the visual search display consisting of a centrally presented picture of a driving scene [from Quétard (2018); Figure 2]^2^. The pictures (29.1 × 21.8 dva), taken from a driver point-of-view, represented a foggy driving scene on a road at night (Colomb et al., 2008). On each picture, the rear lights of a preceding vehicle could be seen (at approximatively 14 m from the driver point-of-view). On some pictures, a child pedestrian (1.4 × 5.1 dva) or a roe deer (3.4 × 3.4 dva) could be present as a target (at approximatively 12 m from the driver point-of-view). The target could be at four possible locations: two external positions (off road, at 10.9 dva to the left or right from the screen center) and two internal positions (on road, at 5.1 dva to the left or right from the center), and was always heading toward the left side of the scene. Half of the pictures were taken under low fog density, while the other half was taken under high fog density (see Quétard, 2018). Each picture contained a smartphone (3.4 × 5 dva) with a gray screen (192, 192, 192; 2.6 × 4.2 dva), displayed at the bottom right corner of the picture, as if put on the car dashboard for being used as a GPS device (the distance between the smartphone center and the right and bottom edges of the picture was, respectively, 3.5 and 2.4 dva). When present, the social reward distractors (i.e., the target circles of the social reward association phase; diameter = 2.4 dva) were displayed at the center of the smartphone’ screen.

Participants performed 384 trials^3^ split equally in 12 experimental blocks, with six consecutive blocks for each fog density level, the order of the fog density levels being counterbalanced across participants. In each block, a target (equiprobably a pedestrian or a roe deer) appeared in 75% of trials. Each target appeared equally often at each of the four possible locations. No target was displayed on the remaining 25% of the trials. Moreover, one-third of the trials contained a high-reward distractor, one-third a low-reward distractor and no distractor was displayed in the remaining third (distractor presence/absence was counterbalanced with target type – child pedestrians, roe deer, none - and position, as well as fog density). Trials order was randomly determined within each block and a short break was offered between each block. Before performing the experimental task proper, participants completed a training block of 12 trials (i.e., stimuli were in same proportion as experimental blocks except that a target was displayed on 50% of the training trials).

Each trial began with a fixation cross displayed for 500, 600, or 700 ms (randomly determined), followed by the driving scene that remained on the screen until the participant’s response (or up to 1,500 ms). He/she had to indicate as fast as possible whether a pedestrian or a roe deer was present on the picture, by pressing “2” or “5” on the numeric keypad with his/her forefinger or middle finger, respectively (the association between the buttons and the target types was counterbalanced across participants). The aim of this discrimination task was to mirror classic attentional task where participants usually have to indicate the orientation of a line (two choices) inside a target (e.g., Anderson et al., 2011b), but also to make the task more difficult and thus introduce more variability in the collected data. No response was required when the target was absent. Consecutively, a feedback screen informed the participants about their accuracy on the current trial (“correct,” “error,” or “missed” – when no response had been given whereas a target was actually present) during 1,500 ms. Finally, a black screen separated two consecutive trials for 500 ms.

## Results

Reaction times (RTs) for incorrect trials or shorter than 200 ms have been excluded from the following analyses. Moreover, for each participant, RTs plus/minus 2.5 absolute deviations around the median (Leys et al., 2013) were also discarded (altogether, less than 5% of the data for both the social reward association and the driving visual search tasks). All analyses relied on linear models, following aggregation at the condition per participant level to make observations independent, hence the need to eliminate extreme RTs that may bias mean estimates. All results were nevertheless confirmed by fitting linear mixed models at trial level, alleviating the need for filtering outlier RTs. The more parsimonious models were reported for readability purpose.

### Social Reward Association Phase

A paired *t*-test comparison revealed that the difference on RTs between high (*M* = 746 ms; SE = 17 ms) and low reward target (*M* = 758 ms; SE = 20 ms) was not significant [*t*_(28)_ = 1.15, *p* = 0.259]. In the same way, error rates were not significantly different for high (*M* = 6.7%; SE = 0.92%) and low reward target (*M* = 7.6%; SE = 1.03%) [*t*_(28)_ = 1.40, *p* = 0.171].

One could expect that an efficient stimulus-reward association should lead to significantly faster and more accurate responses for high reward stimuli. However, although our results were numerically in line with this expectation, our participants were not significantly faster nor more accurate for high versus low reward targets. Nonetheless, as repeatedly observed, significant differences in the association phase are not a necessary pre-requisite to observe value-driven attentional capture in the subsequent search task (e.g., Anderson et al., 2011a; Roper et al., 2014; Anderson, 2016; Anderson and Halpern, 2017; Anderson and Kim, 2019). Non-significant differences could suggest that participants searched for both target colors with roughly equal priority but it does not preclude from investigating whether the experience of these stimulus-reward associations would further influence attention in the test phase, when those stimuli were presented as irrelevant distractors (Anderson, 2016).

### Driving Visual Search Task

Reaction times were submitted to a FoMO × Fog Density (low vs. high) × Distractor Type (high-reward, low-reward, and no distractor) linear model analysis with FoMO as a continuous predictor and Fog Density and Distractor Type as within-participant predictors. Relying on linear models to test the combined effects of factors and continuous predictor, distractor Type was coded using orthogonal Helmert contrasts with the first contrast (C1) opposing RTs on high-reward distractor trials (coded + 1) to RTs on low-reward and no distractor trials (both coded −0.5), and the second (C2) opposing RTs on low-reward trials (coded + 0.5) to RTs on no distractor trials (coded −0.5). Fog was also contrast-coded (low density as −1 and high density as + 1). FoMO scores (*M* = 2.01, *SD* = 0.32) were mean-centered. We predicted that RTs would be significantly longer in the condition of high fog density (indicating that the fog increased attentional demands) and that this effect would be amplified by the interaction of high-reward distractor and FoMO (indicating that distraction occurred).

**FIGURE 3:**
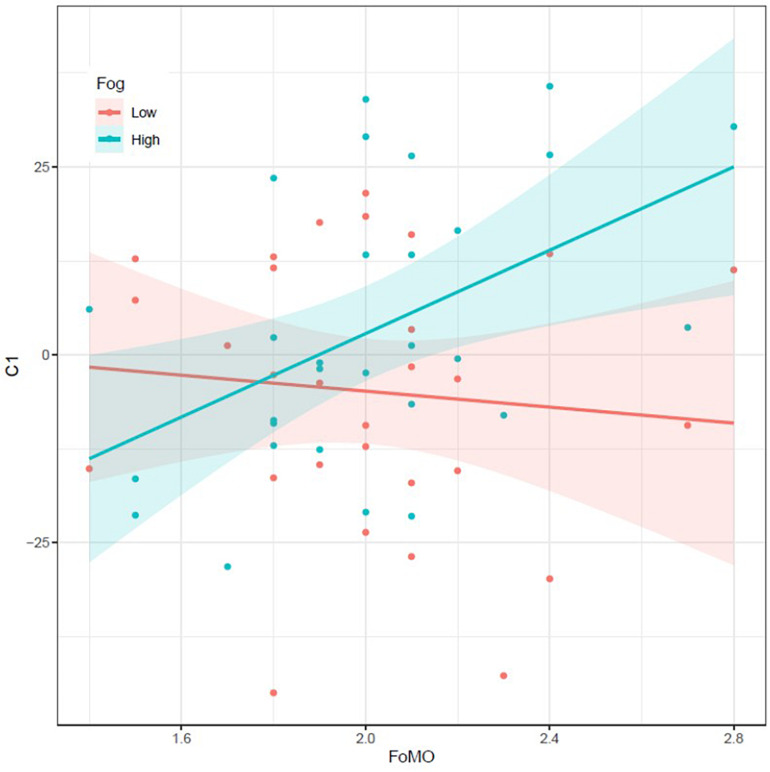
Distraction effect (in ms; with standard error bands) triggered by the high vs. low/no social reward distractor (contrast code C1) under low (red line) and high (green line) fog density for each participant, as a function of Fear of Missing Out (FoMO) scores.

The expected three-way interaction between FoMO, Fog, and Distractor Type (C1) was significant, *b* = 22, 95% CI [3, 41], *t*_(1__08__)_ = 2.31, *p* = 0.023, and *R*^2^ = 0.047^4^. This interaction was decomposed into simple interactions for each level of Fog density. When Fog density was low, the interaction of FoMO and Distractor Type (C1) was non-significant [*b* = −5 ms, 95% CI [−28, 17], *t*_(__27__)_ = 0.5, and *p* = 0.629]. Thus, under low fog density, FoMO played no significant role in the distraction produced by the high-reward distractor. However, and as expected, when the fog density was high (i.e., when attentional demands increased), the interaction of FoMO and Distractor Type (C1) was significant, *b* = 28 ms, 95% CI [7, 48], *t*_(__27__)_ = 2.83, and *p* = 0.009. Indeed, in conditions of high-fog density, high social reward distractors resulted in an increased of RTs by 28 ms for each point on the FoMO scale (Figure 3; *r* = 0.48). In other words, when the fog density was high, higher levels of FoMO were associated with a larger distraction effect for high compared to low reward distractors. The analysis also revealed a main effect of Fog, *b* = 48 ms, 95% CI [33, 63], *t*_(27)_ = 6.61, *p* < 0.001, and *R*^2^ = 0.62, with slower RTs under high (*M* = 731 ms; SE = 18 ms) rather than low fog density (*M* = 683 ms; SE = 17 ms). All other effects and interactions were non-significant. Finally, a similar generalized linear model analysis performed on error rates revealed no significant effect.

## Discussion

The results from the present study revealed that individuals’ level of FoMO can predict the distraction triggered by high social reward stimuli, in a driving context. This observation is in line with previous works showing that participants who are high in FoMO are also those who report paying the most attention to their smartphone when driving (Przybylski et al., 2013; Brown et al., 2021). However, to our knowledge, the present study is the first that relied on direct behavioral observations of distraction, instead of potentially biased self-reported estimations (see Boase and Ling, 2013; Vanden Abeele et al., 2013; Lee et al., 2017).

### Theoretical Implications

To be precise, the link between FoMO and distraction was evident only when the primary task was difficult (i.e., high-fog density). This result is consistent with previous studies showing that smartphones are more likely to elicit distraction under attentional-demanding conditions (Thornton et al., 2014; Stothart et al., 2015). The rationale is that the target search and identification task could have been performed with very little resources in the low fog density. Therefore, attentional capture by reward distractor, if/when present, could not have produced significant performance deficits. One could argue that such interpretation was not strictly supported by our data, as increasing fog density did not significantly increased error rates. However, RTs were significantly longer under high rather than low fog density, suggesting a prolonged accumulating evidence process from visual inputs to reduce decision-making uncertainty (Quétard et al., 2015; Quétard, 2018). This prolonged delay before the participant’s response might offer a larger time window for distractor intrusion (Lavie and de Fockert, 2003). Alternatively, because the fog degraded visual information only outside the vehicle, we could argue that the smartphone and reward distractors were actually relatively more salient in the high rather than low fog density condition. Consequently, along with reward history, higher distractor’s relative salience under high fog density could also have led to larger distraction effect in this condition (see Theeuwes, 1991, 1992; Koch et al., 2013; Zehetleitner et al., 2013).

It therefore appears that FoMO should be taken into consideration when investigating attentional capture by social reward stimuli as it might provide a finer-grained understanding of this phenomenon. Indeed, so far, most studies investigating reward history have associated neutral stimuli with monetary outcomes or points converted into real cash (e.g., Anderson et al., 2011b; Le Pelley et al., 2015). Experimental social reward manipulations are scarcer, probably because they are less meaningful for subjects as they do not have a concrete incidence on their (social) life. We contend that not considering individual characteristics such as FoMO might also account for some null effects (e.g., Johannes et al., 2019). In line with this view, some authors (Wilmer and Chein, 2016) have failed to identify a relationship between mobile engagement and reward sensitivity measured through the classic Behavioral Inhibition/Activation System scale (BIS/BAS: Carver and White, 1994). However, the BIS/BAS scale is not specifically designed for *social* reward sensitivity whereas social aspects are of particular importance when considering smartphone use (Özcan and Koçak, 2003; Srivastava, 2005; Waddell and Wiener, 2014; Sherman et al., 2016). Therefore, we suggest that the FoMO scale would be better suited for such investigations.

### Limitations and Future Research Directions

One important limitation of our study is related to its design. Indeed, our participants were not engaged in a truly realistic nor simulated driving task and thus had no risk of collision with the child pedestrian or the roe deer. As a consequence, the current experimental settings could have been insufficient to incite the participants to strengthen their attentional control settings thereby preventing attentional capture (Gaspelin and Luck, 2018b). Additionally, we did not ask our participants about their driving experience. Because our experimental context was actually relatively different from a real driving activity, one could argue that the participants’ performance might not rely on their driving experience. However, this factor can obviously influence driver behavior and notably, experienced drivers would be more able than novices to respond to hazard and adapt their driving in situation of degraded visibility (e.g., Mueller and Trick, 2012). Therefore, along with FoMO, driver’s experience could be a critical factor to understand smartphone-based distraction and driver’s attentional control under degraded conditions, but more realistic driving scenario and tasks would be necessary. Also, due to our sample characteristics (i.e., mainly young females), it is difficult to generalize our findings to the overall population. Further research is thus needed to clarify the role of FoMO into a more ecological context to offer a better comprehensive model of driver distraction.

Finally, to our knowledge, distractors’ reward history and their motivational values are still underinvestigated in driver distraction studies, with most of the works focusing on salience-based attentional capture [e.g., Chattington et al. (2009), Dukic et al. (2013), but see Walker and Trick (2019) for the impact of emotional irrelevant billboards]. Yet, whereas salience-based distraction can be prevented under some conditions (e.g., Lavie, 1995; Belopolsky and Theeuwes, 2010; Cosman and Vecera, 2010; Gaspelin et al., 2017; Gaspelin and Luck, 2018a, c), attentional capture caused by reward distractors seems to be “automatic” and thus likely to occur in any condition (e.g., Gupta et al., 2016; Munneke et al., 2016; Wang et al., 2018; Matias et al., 2021). Besides, reward-based distraction seems to be qualitatively different from salience-based distraction, as processing reward stimuli hampers a wider range of cognitive process (Anderson, 2017). Hence, attentional capture by smartphone (socially rewarding) notifications is likely to produce effects that persist longer over time (Thornton et al., 2014; Stothart et al., 2015), with more detrimental consequences on drivers’ performances (He et al., 2011; Lemercier et al., 2014). Building more bridges between studies on reward-based and smartphone-based distraction therefore appears of primary importance to improve road safety.

## Conclusion

This preliminary study is the first, to our knowledge, that provides experimental evidences of the link between individuals’ FoMO and social-reward distraction in a driving context. Indeed, we showed that, under difficult (i.e., foggy) driving situations, higher levels of FoMO are associated with larger distraction produced by high social reward stimuli. Therefore, this research emphasizes the necessity for models of road safety to take into account a broader range of drivers’ sources of distraction such as socio-psychological needs, and to go beyond salience-based distraction.

## Data Availability Statement

The raw data supporting the conclusions of this article will be made available by the authors, without undue reservation.

## Ethics Statement

The studies involving human participants were reviewed and approved by CER-IRB UCA. The patients/participants provided their written informed consent to participate in this study.

## Author Contributions

All authors contributed to the study conception, design, statistical analysis, commented on previous versions of the manuscript, read, and approved the final manuscript. JM performed the material preparation, data collection, wrote the first draft of the manuscript.

## Conflict of Interest

The authors declare that the research was conducted in the absence of any commercial or financial relationships that could be construed as a potential conflict of interest.

## Footnotes

1 Note that early studies generally referred to *cell phone* or *mobile phone* instead of *smartphone*, which is, basically, a mobile phone with more advanced features (e.g., browsing the web, emailing, social networking, playing personal media, or games).

2 Original pictures (3,006 × 2,000 pixels) were just trimmed in order to remove irrelevant stimuli for our study (e.g., road signs). To this end, the left, right, and lower edges were, respectively trimmed by 410, 605, and 210 pixels.

3 See https://osf.io/tay95/

4 Partial *R*^2^ standardized effect sizes were estimated using Kenward-Roger approximation of degrees of freedom, following recommendations from Edwards et al. (2008), since we relied on a mixed design with a continuous predictor (FoMO).
