# Supplementary material for: Fear of Missing Out Predicts Distraction by Social Reward Signals Displayed on a Smartphone in Difficult Driving Situations
Source: Front Psychol. 2021 Jul 16;12:688157. doi: 10.3389/fpsyg.2021.688157 (PMC8322628; doi:10.3389/fpsyg.2021.688157)
Supplement: Supplementary file 1 [file Table_1.PDF]

Supplementary Material

Fear of Missing Out scale

Pas du tout exacte	Légèrement exacte	Moyennement exacte	Très exacte	Parfaitement exacte
1	2	3	4	5

a) Je crains que les autres vivent des expériences plus gratifiantes que les miennes.
I fear others have more rewarding experiences than me.

b) Je crains que mes amis vivent des expériences plus gratifiantes que les miennes.
I fear my friends have more rewarding experiences than me.

c) Je suis préoccupé·e ou inquiet·ète lorsque j'apprends que mes amis sont en train de s'amuser sans moi.
I get worried when I find out my friends are having fun without me.

d) Je deviens anxieux·se quand je ne sais pas ce que mes amis sont en train de faire.
I get anxious when I don't know what my friends are up to.

e) Il est important pour moi de comprendre les blagues qui circulent dans mon groupe d'amis.
It is important that I understand my friends "in jokes".

f) Parfois, je me demande si je passe trop de temps à me tenir informé·e de ce qui se passe en ce moment.
Sometimes, I wonder if I spend too much time keeping up with what is going on.

g) Cela m'ennuie quand je manque une opportunité de voir mes amis.
It bothers me when I miss an opportunity to meet up with friends.

h) Quand je passe un bon moment, il est important pour moi de partager les détails en ligne, sur les réseaux sociaux (par exemple, en mettant à jour mon statut).
When I have a good time it is important for me to share the details online (e.g. updating status).

i) Quand je manque une soirée ou une sortie, cela me dérange.
When I miss out on a planned get together it bothers me.

j) Quand je pars en vacances, je continue de garder un œil sur ce que mes amis sont en train de faire.
When I go on vacation, I continue to keep tabs on what my friends are doing.

Supplementary Figure 1. French version of the Fear of Missing Out scale, retrieved from Michot et al. (2016). For each of the 10 affirmations, participants were asked to say whether it may applied to them by using a 5-points Likert scale, ranging from 1 " Not at all true for me" to 5 "Perfectly true for me".

Material and method of the social reward association phase

The social reward association was achieved through a classic visual search task (adapted from Anderson, 2016; Figure 1). First, a fixation screen contained a black cross centrally displayed on a gray background (RGB: 192, 192, 192). For the visual search screen, six colored circles (diameter = 2.4 degree of visual angle, dva) were uniformly distributed around the central black cross, on an imaginary circle, with two circles vertically aligned with the cross. The center-to-center distance between a circle and the cross or between two adjacent circles was 4 dva. For each participant, two colors among blue (0, 0, 255), green (0, 175, 0) or red (255, 0, 0) were selected as target colors (counterbalanced across participants). The target contained equally often a vertical or horizontal black line (1.1 x 0.1 dva) in its center whereas non-target circles contained a tilted black line (45° to the left or right, randomly determined). The colors for the five non-target circles were orange (255, 130, 0), cyan (0, 255, 255), gold (255, 215, 0) or purple (255, 0, 255) plus the remaining unselected target color (e.g., blue, green or red).

The association between target colors and social (low or high) rewards was achieved through the presentation of a neutral or happy face during the feedback trial (Anderson, 2016). Twelve models (6 females, 6 males) were retrieved from the Radboud Faces database (Langner et al., 2010). For each model, a neutral and a happy face picture were selected, representing 24 pictures in all (for face characteristics see Supplementary Table 1). For each participant, one target color was associated with a low social reward outcome whereas the other color was associated with a high social reward (color-reward association counterbalanced across participants). To this end, a neutral face was displayed during feedback on 80% of correct trials while a happy face appeared on the remaining 20%, for the low social reward association. Conversely, a happy face was displayed on 80% of correct trials and a neutral face on the remaining 20%, for the high reward association. Models for neutral and happy faces were randomly selected on each trial.

Participants performed 240 trials[1] split equally in five experimental blocks. For each block, in which trial order was randomly determined, half of the trials contained a high-reward target, whereas a low-reward target appeared for the other half. Participants first performed a training block containing 12 trials. A short break was allowed between each block. A trial began with a fixation cross displayed during 500, 600 or 700 ms (randomly determined). Then, the visual search screen was displayed until the participant's response or 1500 ms maximum. The colored target appeared equally often at each of the six possible locations and the five non-target circles were displayed randomly at the remaining locations. Participants were asked to report the orientation (i.e., horizontal or vertical) of the line inside the target, by pressing "b" or "n" on the keyboard with their right forefinger and right middle finger respectively. The association between response buttons and line orientation was counterbalanced across participants. Thereafter, a feedback was prompted during 1500 ms, indicating the participant's accuracy (i.e., "correct", "error" or "missed" in case of no response), along with the neutral or happy face for correct trials. Finally, a grey screen separated two consecutive trials for 500 ms.

[1] See https://osf.io/tay95/

Supplementary Table 1. Characteristics of faces selected from the Radboud Faces Database (Langner et al., 2010). Abbreviations for columns are as follows: %Agr: percentage of agreement on emotion categorization; Int: mean intensity rating for the facial expression; Clar: mean clarity for the facial expression; Gen: mean genuineness for the facial expression; Val: mean valence rating for the image; Attract: average attractiveness ratings.

Model	Sexe	Emotion	%Agr	Int	Clar	Gen	Val	Attract
7	male	happy	100	4,63	4,75	3,5	4,42	2,8
7	male	neutral	100	3,71	4,25	4,17	3,5	2,8
15	male	happy	100	4,00	4,15	3,95	4,25	2,6
15	male	neutral	100	3,62	4	4,14	3,33	2,6
19	female	happy	100	4,08	4,73	4,38	4,58	2,7
19	female	neutral	88	3,62	3,58	3,96	2,92	2,7
23	male	happy	100	4,14	4,45	3,95	4,5	2,7
23	male	neutral	100	4,09	4,3	4,17	3,43	2,7
26	female	happy	100	4,00	4,46	3,54	4,21	2,7
26	female	neutral	100	3,58	3,71	3,67	3,25	2,7
30	male	happy	100	4,22	4,48	4	4,17	2,9
30	male	neutral	92	3,54	3,54	4,21	3,33	2,9
31	female	happy	100	3,87	4,43	4,35	4,39	2,6
31	female	neutral	96	3,61	3,74	4,22	3,35	2,6
32	female	happy	96	3,92	4,54	4,42	4,5	2,5
32	female	neutral	100	3,58	3,96	3,96	3,08	2,5
33	male	happy	100	4,24	4,56	4,04	4,48	2,5
33	male	neutral	100	3,92	4,04	3,96	3,21	2,5
58	female	happy	100	3,62	4,38	3,76	4,29	2,4
58	female	neutral	86	3,36	4	4,23	3,05	2,4
61	female	happy	100	4,18	4,36	2,59	4,23	2,7
61	female	neutral	91	3,52	4,09	4,35	3,17	2,7
71	male	happy	100	4,71	4,76	4,05	4,67	2,8
71	male	neutral	100	3,55	4,15	4,2	3,5	2,8

Importantly, the valence rating between happy (*M* = 4.4; *SD* = 0.16) and neutral faces (*M* = 3.3; *SD* = 0.18) is significantly different [$t(11) = 16.6$, $p < .001$]. Additionally, happy faces (*M* = 4.1; *SD* = 0.3) are perceived as more intense [$t(11) = 5.48$, $p < .001$] than neutral faces (*M* = 3.6; *SD* = 0.19). Clarity is also higher [$t(11) = 6.4$, $p < .001$] for happy (*M* = 4.5; *SD* = 0.19) than neutral faces (*M* = 3.9; *SD* = 0.25) but no significant difference is observed for genuineness nor agreement [$ts(11) < 2.16$, *ns*]. Note that attractiveness ratings are model-based rather than emotion-based, thus the same scores are entered for happy and neutral faces for a given model.
